# Fabrication of High-Performance Natural Rubber Composites with Enhanced Filler–Rubber Interactions by Stearic Acid-Modified Diatomaceous Earth and Carbon Nanotubes for Mechanical and Energy Harvesting Applications

**DOI:** 10.3390/polym15173612

**Published:** 2023-08-31

**Authors:** Md Najib Alam, Vineet Kumar, Han-Saem Jung, Sang-Shin Park

**Affiliations:** School of Mechanical Engineering, Yeungnam University, 280, Daehak-ro, Gyeongsan 38541, Republic of Korea; mdnajib.alam3@gmail.com (M.N.A.); vineetfri@gmail.com (V.K.); depictme@naver.com (H.-S.J.)

**Keywords:** rubber nanocomposites, diatomaceous earth, carbon nanotubes, mechanical properties, electrical properties, energy harvesting

## Abstract

Mechanical robustness and high energy efficiency of composite materials are immensely important in modern stretchable, self-powered electronic devices. However, the availability of these materials and their toxicities are challenging factors. This paper presents the mechanical and energy-harvesting performances of low-cost natural rubber composites made of stearic acid-modified diatomaceous earth (mDE) and carbon nanotubes (CNTs). The obtained mechanical properties were significantly better than those of unfilled rubber. Compared to pristine diatomaceous earth, mDE has higher reinforcing efficiencies in terms of mechanical properties because of the effective chemical surface modification by stearic acid and enhanced filler–rubber interactions. The addition of a small amount of CNT as a component in the hybrid filler systems not only improves the mechanical properties but also improves the electrical properties of the rubber composites and has electromechanical sensitivity. For example, the fracture toughness of unfilled rubber (9.74 MJ/m^3^) can be enhanced by approximately 484% in a composite (56.86 MJ/m^3^) with 40 phr (per hundred grams of rubber) hybrid filler, whereas the composite showed electrical conductivity. At a similar mechanical load, the energy-harvesting efficiency of the composite containing 57 phr mDE and 3 phr CNT hybrid filler was nearly double that of the only 3 phr CNT-containing composite. The higher energy-harvesting efficiency of the mDE-filled conductive composites may be due to their increased dielectric behaviour. Because of their bio-based materials, rubber composites made by mDE can be considered eco-friendly composites for mechanical and energy harvesting applications and suitable electronic health monitoring devices.

## 1. Introduction

Rubber composites have attracted considerable attention in recent technological applications [1]. Rubber composites have advantages over other polymer composites, such as high stretchability, low glass transition temperatures, excellent resiliency, and good abrasion resistance. These properties make rubber composites suitable for applications in tyres, tubes, conveyor belts, and shoes. Owing to their stretchability, rubber composites are used in many advanced applications, such as electromagnetic interference shielding [2,3,4], strain sensors [5,6,7], nanogenerators [8,9], and other stretchable devices [10,11,12].

Fillers play a vital role in determining the functionality of rubber composites. Depending on their reinforcement capability, fillers can be classified as reinforcing, semi-reinforcing, or non-reinforcing. Non-reinforcing fillers are used to reduce the cost of rubber composites. However, proper modification of these fillers has changed them to semi-reinforcing or even reinforcing fillers, which can improve certain mechanical properties of rubber composites [13]. Reinforcing fillers are generally on the nanometer scale and have a large interacting surface area that can strongly bind rubber chains to their surface. Silica and carbon black are the two most abundant fillers used in the tyre industry for mechanical reinforcement. Carbon nanomaterials interact very well with rubber, followed by van der Waals and π–π stacking interactions [14]. Among different carbon nanomaterials, carbon nanotubes (CNTs) are highly useful for improving the mechanical, thermal, and electrical properties of rubber composites [15]. Because of their one-dimensional rod-like morphology, high electrical conductivity (>10^2^–10^6^ S/cm), and high thermal conductivity (>2000 Wm^−1^ K^−1^), a small amount of CNT can significantly enhance the electrical and thermal properties of rubber compounds.

Rubbers are dielectric materials with negligible or very low electrical conductivity. Conducting materials must be incorporated to make rubber a conducting material. A wide range of conducting rubber composites can be fabricated depending on the structure and conductivity of the filler. In addition to their mechanical applications, conducting rubber composites have gained wide attention in modern electronic devices [16,17]. Owing to their mechanical robustness, conducting rubber composites can replace metal-based conductors, in which stretchability is a vital factor. Piezoresistive and piezoelectric behaviours of rubber composites have been observed in various applications such as sensors, actuators, and electromechanical transducers [18,19]. Electrical power generation using dielectric elastomers is highly renewable and has gained popularity in recent years [20,21,22]. Dielectric materials play a significant role in capacitance-based energy harvesting devices. Rubber has a very low dielectric loss and is suitable for energy harvesting at low frequencies. To further improve the dielectric constant, the addition of ceramic materials with high dielectric constants is useful [23,24]. However, these materials should be used in higher proportions to achieve the percolation of polymer composites [24]. Salaeh et al. [25] used barium titanate and lead titanate to improve the dielectric behaviour of natural rubber. Although these ceramic materials provide improved dielectric rubber composites at higher proportions, they significantly reduce the mechanical properties of the composite and enhance the hysteresis loss. Moreover, ceramic materials are expensive, and heavy-metal-based ceramics may be toxic. Hence, energy-harvesting composites with cheap and nontoxic filler materials may be of great interest for energy-harvesting devices, especially health-monitoring self-sensing devices.

Recently, biofillers have become popular in rubber compounds [26]. They are attractive because of their low cost, natural availability, non-toxicity, and hierarchical structure. Among the different natural fillers, natural fibres, crystalline cellulose, bone dust, biochar, and bio-calcium carbonate have been successfully applied in rubber compounding, either in pristine or modified forms [27]. From the viewpoint of toxicity and the limitations of petroleum resources, scientists have tried to establish silica as an alternative filler to carbon black in the tyre industry, with some special advantages [28,29,30]. Silica fillers mainly reduce the rolling resistance of tyres, which reduces the fuel cost of vehicles [30]. Although silica filler partially or fully replaces carbon black, depending on the rubber products with reduced toxicity, synthetic silica filler remains costly in rubber compounding, as it requires some critical steps [30].

In addition to synthetic silica, biogenic silica can serve as an alternative to silica resources. Beidaghy et al. [31] discussed different procedures for producing silica from rice husk and straw. Choophun et al. [32] successfully utilised rice husk-derived silica for rubber compounding, which may have applications in tyre tread formulations. Recently, Barrios and colleagues [33] have discussed various sustainable fillers, such as chitin, chitosan, lignin, and cellulose, for elastomeric compounds. Sustainable fillers might possess a high content of polar groups that are not compatible with non-polar rubber. Additionally, they contain a high level of absorbed moisture that inhibits the interfacial interactions between the filler and rubber [34]. Rubber–filler interactions can be enhanced with these filler systems, followed by drying the filler at an elevated temperature and surface modification. Occasionally, the methods are not straightforward or inexpensive, which has hindered the industrial applications of these fillers in rubber compounding. Diatomaceous earth is another major source of biogenic silica [35]. The diatomaceous earth comprises the focal remains of diatoms (hard-shelled microalgae). It mainly consists of 80–90% silica, 2–4% alumina, and 0.5–2% iron oxide. In a review paper, Reka et al. [36] discussed diatomaceous earth in detail, along with its different applications. They discovered that diatomaceous earth had a low level of moisture content that could be easily removed by heating and exhibited good thermal stability. Diatomaceous earth naturally contains hierarchical structures because of the hard-shelled structure of diatoms. Filler morphology plays an important role in rubber reinforcement [37]. Higher filler structures may improve the mechanical strength of rubber compounds, owing to the contribution of filler–filler mechanical interactions [38]. Diatomaceous earth can also be used to improve the dielectric properties of rubber [39]. It also contains certain metal oxides that can participate as cure activators in sulphur vulcanisation [40]. However, despite possessing several advantageous properties for use as a filler in rubber compounding, diatomaceous earth has drawbacks associated with polar functional groups on its surface. Because diatomaceous earth is a naturally abundant, cheap material with a highly porous structure, it is used as a filler in different rubber composites [41,42,43,44,45]. 

Among different types of rubber, natural rubber is the most widely used in industrial products. Some benefits, such as abundant natural availability, non-toxicity, low cost, excellent resilience, high stretchability, and good scratch resistance, can only be observed in natural rubber [46]. Liliane Bokobza [47], Sethulekshmi et al. [48], and Sethulekshmi et al. [48] have reviewed natural rubber-based composites with numerous filler systems. Natural rubber shows very good mixing behaviour with all types of fillers and enhances the desired properties [47,48]. Inorganic nanofillers or surface-modified fillers have a greater effect on the reinforcement of rubber properties. Similar to other clay minerals, diatomaceous earth can also be used as a reinforcing filler in rubber composites. However, owing to the larger particle size and polarity, it remains challenging to improve the tensile properties other than the stiffness of rubber composites. Although there are many possibilities for enhancing the properties of rubber composites with pure or modified diatomaceous earth, only a few reports have been published [41,42,43,44,45,49]. To the best of our knowledge, this is the first study on diatomaceous earth and carbon nanotubes as hybrid reinforcing fillers in natural rubber composites for suitable applications in mechanical and self-powered electromechanical sensing.

In this study, we aim to fabricate novel natural rubber composites comprising stearic acid-modified diatomaceous earth and carbon nanotubes. Motivated by the excellent oil absorption properties of diatomaceous earth, a simple and efficient method was developed to alter its polarity using stearic acid. Thus, the conversion of the polar surface to the non-polar surface of diatomaceous earth could significantly improve the mechanical properties of natural rubber by improving the interfacial interactions. Because the mechanical stability of rubber composites is the most important factor for all types of stretchable applications, we mainly discuss the mechanical properties of rubber composites, along with some basic electrical properties. After the successful fabrication of rubber composites using modified diatomaceous earth and CNT-based hybrid filler systems, the energy-harvesting performances of the composites were evaluated and discussed.

## 2. Materials and Methods

### 2.1. Materials

An ultra-soft masterbatch rubber compound was prepared by mixing the natural rubber (STR 5L), zinc oxide, stearic acid, accelerator tetramethyl thiuram disulfide, accelerator N-tert-butyl-2-benzothiazolimesulfonamide, and sulphur in 100, 5, 2, 1, 1.75, and 1.5 amounts in gram, respectively, in a two-roll mill for about 30 min. The rubber and curing ingredients were sourced from local rubber goods manufacturing companies, Gyeongsang, Republic of Korea. Multi-walled carbon nanotubes (specific surface area: ~250 m^2^/g) were obtained from Hanwha Nanotech Corporation, Seoul, Republic of Korea. Good quality filtration-grade diatomaceous earth (DE) powder was purchased from Sigma–Aldrich (St. Louis, MO, USA). Stearic acid was purchased from Sigma–Aldrich (USA). Toluene was purchased from Daejung Chemicals & Metals Ltd., Siheung, Republic of Korea.

### 2.2. Preparation of Masterbatch Rubber 

The raw rubber was initially masticated on a two-roll mill for approximately 20 min. Subsequently, zinc oxide and stearic acid were incorporated and milled for about 5 min. Lastly, accelerators and sulphur were added simultaneously and milled for another 5 min before being removed from the mill. The parameters and fabrication method for the masterbatch rubber can be located elsewhere [40].

### 2.3. Stearic Acid Treatment of Diatomaceous Earth

First, 2 g of stearic acid was dissolved in 100 mL of toluene via ultrasonication for a few minutes. After that, 20 g of pristine DE was added to the solution and kept for 30 min in an ultra-sonication bath at 25 °C. After sonication, the colloidal-like mixture was dried at 80 °C for complete drying. The dried compounds (mDE) were stored in a desiccator. The ratio of diatomaceous earth to stearic acid for filler modification was fixed at 10:1 (*w*/*w*). Typically, coupling agents below 10 wt% were employed in relation to the silica filler based on the quantity of functional groups in terms of molar content [28].

### 2.4. Characterizations of Fillers

The morphologies of the filler and tensile fractured rubber sections were characterised by field-emission scanning electron microscopy (FE-SEM, S-4800, Hitachi, Tokyo, Japan). Before the SEM analysis, the samples were sputter-coated with platinum. The diatomaceous earth was characterised before and after treatment with stearic acid using Fourier transform infrared spectroscopy (FT-IR) to investigate the chemical changes in the functional groups at 4 cm^−1^ resolution and two scan numbers.

### 2.5. Fabrication of Rubber Composites

The solvent-bending method was applied to fabricate rubber composites by maintaining filler structures similar to their pristine forms. First, 25 g of masterbatch rubber was soaked in 150 mL of toluene in a glass jar for one day. A smooth rubber slurry was obtained via mechanical stirring. In another vessel, the required amount of filler(s) was added to 100 mL of toluene and sonicated for approximately 30 min. The two slurries were mixed and vigorously stirred for 10 min. The final slurry was then transferred to a flat tray and dried in an oven at 80 °C. It should be noted that appropriate handling and reutilisation of the solvent (toluene) can have a negligible environmental impact. Furthermore, achieving a nano-level filler distribution is only feasible through solvent blending methods. Nevertheless, an excessive use of solvent can lead to the sedimentation of the filler particles. The conventional dry mixing on the two-roll mill was avoided because it could disrupt the anisotropy of the filler particles and potentially result in the loss of electrical conductivity in the rubber composite. The advantages of employing the solvent-blending technique can be found elsewhere [50,51]. The dried compounds were vulcanised in a hot press at 150 °C for 15 min as sheets, cylindrical samples, and electrodes, as previously described [50,51]. Details of the mixing compositions are listed in Table 1.

### 2.6. Mechanical Properties

Compressive mechanical properties were evaluated using cylindrical samples (d = 20 mm × h = 10 mm), and tensile mechanical properties were evaluated using dumbbell-shaped (ISO-37, Type-2) [52] test pieces in a universal testing machine (Lloyd, Westminster, UK) using a 1 kN load cell. The deformation rates for the compressive and tensile tests were 2 mm/min and 300 mm/min, respectively. The load-carrying capacities of the rubber composites after multiple cycles were determined using dimensions identical to those of the cylindrical samples. Different mechanical properties, such as Young’s modulus, modulus at 10% elongation, tensile strength, elongation at break, and fracture toughness, were obtained from the stress–strain data, and their average values are presented. Four specimens were scrutinised for each composition, and the most representative stress–strain data were presented.

### 2.7. Swelling Properties

To determine the solvent swelling index and cross-link density in the rubber composites, cylindrical samples of the above dimensions were kept in toluene for 7 days to reach equilibrium. After 7 days, the surface toluene was immediately removed using blotting paper, and the swollen weight was measured. The swelling index was calculated as follows from Equation (1):

(1) $$Swelling\;Index=\frac{\left(Swelled\;weight-Initial\;weight\right)}{Initial\;weight}$$

The chemical cross-link densities of the vulcanised compounds were calculated from the equilibrium swelling data according to the Flory–Rehner equation [53] as presented in Equation (2):

(2) $$V_{c}=-\frac{\left\{ln\left(1-V_{r}\right)+V_{r}+\chi V_{r}^{2}\right\}}{\left\{V_{s}d_{r}\left(V_{r}^{\frac{1}{3}}–\frac{V_{r}}{2}\right)\right\}}$$

where V_c_ is the cross-link density of the rubber vulcanisate, V_r_ is the volume fraction of rubber in the swollen compound, χ = 0.3795 is the interaction parameter of the natural rubber and toluene system, V_s_ = 106.2 cm^3^/mole is the molar volume of toluene (solvent), and d_r_ is the density of the rubber vulcanisate.

The volume fractions of rubber compounds were calculated by this formula in Equation (3),

(3) $$V_{r}=\frac{\left(\frac{w_{r}}{d_{r}}\right)}{\left\{\left(\frac{w_{r}}{d_{r}}\right)+\left(\frac{w_{s}}{d_{s}}\right)\right\}}$$

where w_r_ is the weight of the rubber taken, dr is the density of the rubber vulcanisate, which was obtained by the formula density = mass/volume considering the cylindrical sample (d = 20 mm × h = 10 mm), w_s_ is the weight of the swollen solvent, and d_s_ = 0.87 g/cm^3^ is the density of the swollen toluene (solvent).

### 2.8. Electrical and Electromechanical Sensing Properties

The electrical resistivity was calculated by this formula in Equation (4),

(4) $$\rho =\frac{RA}{L}$$

where ρ is the electrical resistivity, R is the electrical resistance of the rubber composite, A is the area of the electrode, and L is the distance between the electrodes. The resistance (R) was measured using a source meter with copper electrodes placed on opposite sides of the cylindrical sample (d = 20 mm × h = 10 mm).

The electrical conductivity of the rubber composites was found by this formula in Equation (5),

(5) $$Conductivity=\frac{1}{\rho }$$

Electromechanical energy-harvesting devices were prepared by placing the composites as 1 mm thick electrodes on opposite sides of a 5 mm thick unfilled rubber sheet. The electromechanical activity of the rubber composites in energy-harvesting systems was measured using the output voltages. The electrodes were connected to a source metre (Agilent, Model: 34401A, Santa Clara, CA, USA), and a dynamic load of 50 kPa was applied to the top of the DC electrode using a loading tip. During cyclic loading–unloading, the energy-harvesting device exhibited changes in the output voltages. At similar mechanical loads, the voltage output can be considered the electromechanical energy-harvesting efficiency of these composites.

## 3. Results and Discussion

### 3.1. Characteristics of the Filler Materials

**Figure 1:**
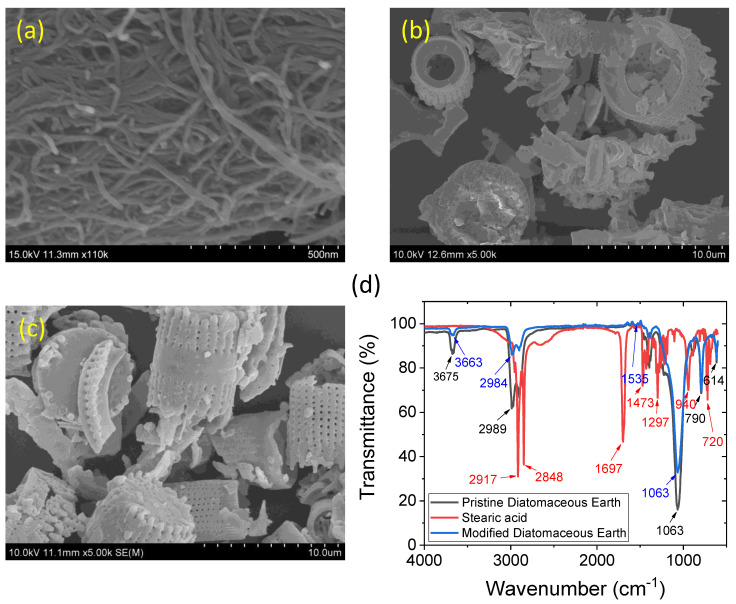
SEM images of (**a**) multi-walled carbon nanotube, (**b**) pristine diatomaceous earth, and (**c**) stearic acid-modified diatomaceous earth; (**d**) FT-IR spectroscopies of pristine DE (black line), stearic acid (red line), and modified DE (mDE) (blue line).

The morphology of the CNTs and pristine DE can be seen in Figure 1a,b. From Figure 1a, it can be seen that the diameters of the CNTs are less than 50 nm, and the lengths vary from a few hundred nanometers to micrometres. The SEM images suggest that the CNTs have a higher aspect ratio. Because of the high aspect ratio of the CNT, a small amount was sufficient to achieve electrical percolation in the rubber composite. Figure 1b suggests that the DE particles are micrometres in size and have hierarchical porous structures. Owing to the porous structures and cavities inside the DE, rubber molecules can easily enter the particles, and effective reinforcement is possible because of physical interactions. However, owing to the higher polarity of DE, chemical interactions are expected to be poor between unmodified DE and non-polar rubber molecules. Figure 1c shows the morphology of the stearic-modified DE. It is difficult to visualise the surface modification of DE with a morphology similar to that of untreated DE. Hence, stearic acid may have better chemical interactions than film formation on the DE surface. The detailed chemical interactions between stearic acid and DE were ascertained from the FT-IR spectra shown in Figure 1d.

The black line in Figure 1d represents the untreated DE. The strong band at 3675 cm^−1^ may arise from the in-phase symmetric vibration of OH groups on either the outer or inner surface of the octahedral sheets that are weakly hydrogen-bonded with the next tetrahedral sheet, as found in kaolinite [54,55]. Because DE contains kaolinite structures, it is reasonable to determine this band. The peak at 2989 cm^−1^ may be due to unknown organic impurities in the DE. The 1063 cm^−1^ band could be attributed to a Si-O-Si stretching vibration [54]. The band at ~790 cm^−1^ was assigned to the OH translational vibration [54,56].

The red line in Figure 1d represents the FTIR spectrum of stearic acid. The peaks at approximately 2917 and 2848 cm^−1^ are representative of the stretching vibrations of the CH_2_ groups [57]. The peak at ~1697 cm^−1^ represents the stretching of the stearic acid carbonyl group. From the observed spectra of the modified DE (blue line), it can be seen that the peak of the carbonyl group was completely absent, although the modified DE was not washed to remove the unreacted stearic acid. The absence of this peak strongly indicates the conversion of carbonyl to carboxyl anions through chemical bonding with DE, which results in a new peak at 1535 cm^−1^ for the antisymmetric stretching vibration of the C=O bond in the carboxyl group [57,58]. It is believed that, in addition to silica and kaolinite in DE, there are many basic materials [36,54] that react with stearic acid and form strong bonds. Because of these strong interactions, the intensities of the other functional groups in DE were either suppressed or tended to be lower when we compared the black and blue lines of the pristine and modified DE, respectively, as shown in Figure 1d.

**Figure 2:**
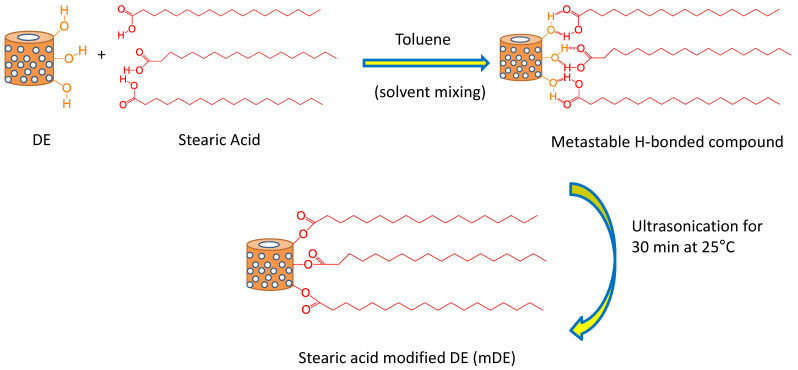
Schematics for the modification of diatomaceous earth by stearic acid.

The mechanism of action of stearic acid medication is illustrated in Figure 2. It is believed that stearic acid molecules dissolved in toluene can attach to the DE surface via H bonding and form a metastable compound [59]. Upon ultrasonication, the metastable compound finally formed a stable modified compound, followed by a condensation-type chemical reaction [58]. It is believed that, upon vibration, the gallery between the octahedral and tetrahedral sheets may expand and become feasible for exchanging the hydroxyl group from the octahedral site with the carboxyl group from stearic acid. At a 10:1 ratio of DE to stearic acid, there may be a complete condensation reaction between DE and stearic acid because the stearic acid peaks for the carbonyl group are absent in the modified DE. Thus, stearic acid modification can considerably lower the surface polarity of DE and can interact strongly with non-polar natural rubber, as evidenced by the various properties in later sections.

### 3.2. Mechanical and Physical Properties of the Rubber Composites

**Figure 3:**
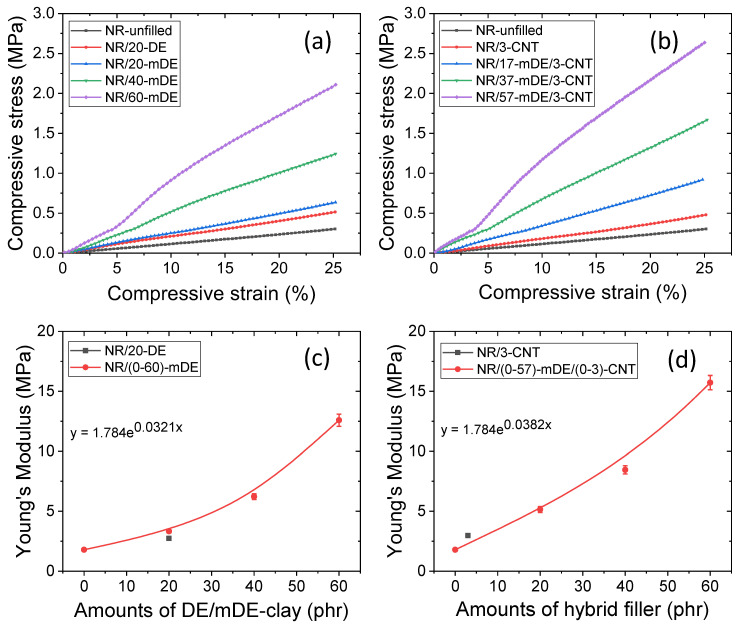
Compressive mechanical properties, (**a**,**b**) compressive stress–strain curves, and (**c**,**d**) Young’s modulus as a function of filler amounts.

Various compressive mechanical properties are shown in Figure 3a–d. From the compressive stress–strain curves in Figure 3a, it is clear that the mDE-filled compound (NR/20-mDE) has a better compressive modulus than the pristine DE-filled (NR/20-DE) compound. This could be due to additional interactions between the rubber and modified DE (mDE). In addition, the compressive modulus increased with increasing mDE content (Figure 3a). Interestingly, after 40 phr of mDE, the stress–strain slope lost linearity at a higher compressive strain. This could be due to filler percolation above 40 phr DE. The clear slope change of approximately 5% of the compressive strain in the 60 phr mDE-containing composite (NR/60-mDE) may be due to strain-dependent filler percolation. However, with up to 5% compression, the stress–strain slope remained linear. Hence, mDE can be useful as a filler for up to 60 phr when low deformation and high compressive strength are required. The addition of CNTs to the mDE-filled compounds further increased the compressive modulus (Figure 3b). This could be due to the improved filler dispersion in the hybrid filler systems and the higher reinforcing power of the CNT fillers. The highest compressive modulus is obtained for the NR/57-mDE/3-CNT system. The variation in Young’s modulus with the amount of filler is plotted in Figure 3c,d. From these figures, it can be observed that the increase in Young’s modulus with the filler amount is more exponential for the hybrid filler systems than for the mDE-only filler systems. This suggests that overall interactions combining physical and chemical hybrid filler systems have a greater value than mDE-filled compounds at similar filler amounts. This may be due to the higher aspect ratio and higher interfacial interactions between the rubber and CNT [60].

**Figure 4:**
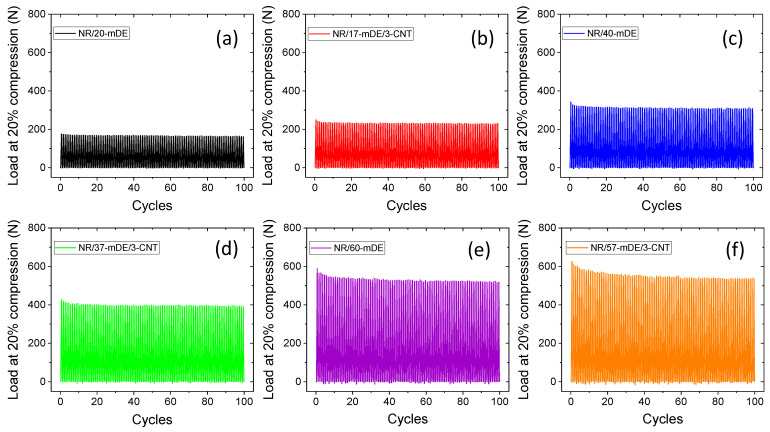
Effect on compressive load at 20% deformation with loading–unloading cycles in different rubber composites: (**a**) NR/20-mDE, (**b**) NR/17-mDE/3-CNT, (**c**) NR/40-mDE, (**d**) NR/37-mDE/3-CNT, (**e**) NR/60-mDE, and (**f**) NR/57-mDE/3-CNT.

Cyclic compressive loading–unloading tests were performed to study actual dynamic compressive mechanical applications. The results are shown in Figure 4a–f. From these figures, it can be concluded that the hybrid filler systems have a higher load-carrying capacity than the mDE filler systems with similar filler amounts. Interestingly, for the mDE-only filler systems, the load value stabilised after approximately 5–10 cycles. However, the load stabilised at higher cycles for the hybrid filler systems. It can be seen that the load-carrying capacity is significantly higher up to 40 phr of filler amounts in the hybrid filler systems compared to mDE filler systems only at similar filler amounts. From Figure 4e,f, it can be observed that the load value decreases with increasing cycles. However, stable load values were obtained after 50 cycles at higher filler loadings. The decreasing load-carrying capacity was due to hysteresis loss, which might be due to the permanent breakdown of some filler networks and stress softening [61]. The better load-carrying capacity, even after many cycles, indicates that mDE-based rubber composites can be useful for compressive applications, even at higher filler loadings.

**Figure 5:**
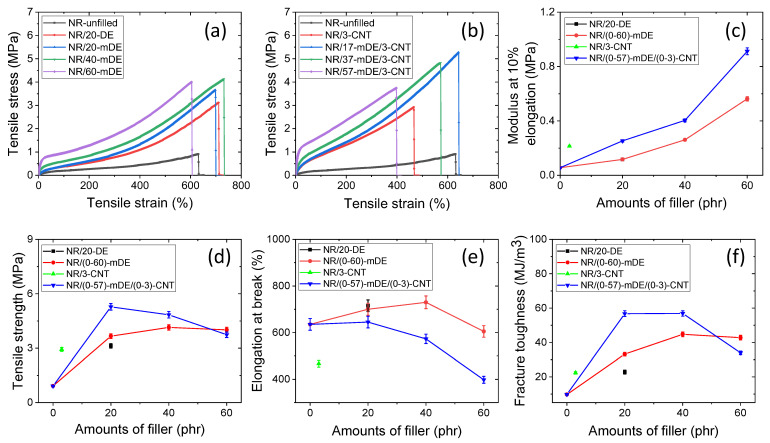
Tensile mechanical properties: (**a**,**b**) stress–strain curves, (**c**) modulus at 10% deformation, (**d**) tensile strength, (**e**) elongation at break, and (**f**) fracture toughness.

Figure 5a–f shows the various tensile mechanical properties. From the curves in Figure 5a,b, it is evident that the stress–strain relationships are hyperelastic. With increasing filler content, the stress–strain curve became more nonlinear at lower strains. At the beginning of the curve, a higher slope value indicates the existence of filler–filler interactions that inhibit extension at lower deformations [38]. With increasing filler amounts, the filler–filler interactions become more prominent. After a critical distance of nearly 10% elongation, the filler–filler interactions were minimised. Above this deformation, the stress–strain slopes better represented the filler–polymer and polymer–polymer interactions [38]. According to the lower stress–strain slope of the unfilled rubber, it is difficult to say that stress-induced crystallisation occurs in these vulcanised systems. The addition of filler greatly improved the stress–strain slope value, which can be attributed to the improved filler–filler and filler–polymer interactions in the rubber composites.

Because the modulus at a small deformation better indicates filler–filler interactions [38], the modulus value at this deformation can provide information regarding the filler percolation threshold. From Figure 5c, the greater change in the modulus from 40 to 60 phr could be due to filler percolation above 40 phr. Hybrid fillers show a higher change in modulus with filler amounts, which could be due to the improved filler distribution and anisotropic structure of the CNT, which produces hybrid filler networks [62,63].

Figure 5d shows the variation in tensile strength with the filler amount. From this figure, it can be understood that the tensile strength was significantly improved at 20–40 phr filler amounts for the unfilled rubber. This may be due to the formation of a sufficient amount of the rubber–filler network at this filler level. The highest tensile strength was obtained in the hybrid filler system (NR/17-mDE/3-CNT) with 20 phr of filler. This further suggests that the hybrid filler showed improved filler distribution and reached filler percolation at lower filler amounts. Up to a 40 phr filler amount, both filler systems showed better tensile strength, but beyond that amount, the tensile strength decreased more rapidly with increasing filler content. It was observed that with a hybrid filler system at 40 phr, the tensile strength was approximately 429% higher compared to the unfilled rubber vulcanisate. A similar observation was noted in the case of a silica–kaolinite mixed mineral filler, similar to diatomaceous earth, where after 40 phr of filler, there were no significant changes in the tensile strength values [13]. At higher filler amounts, the filler–filler interactions were enhanced at the expense of the filler–polymer interactions, owing to the lowering of the rubber fraction. It was found that similar mDE filler amounts provided better tensile strengths than the pristine ones. This suggests that the modified DE improved the filler–polymer interactions. Significant improvements in the tensile strength of the hybrid filler systems were also possible because of the higher surface area of the CNT.

Figure 5e shows the variation in the elongation at break with respect to the filler amount. From this figure, it can be seen that the elongation at the break can be enhanced by the addition of up to a critical amount of filler. This strongly supports the formation of filler–polymer networks at the filler percolation level that are more flexible and reversible than chemical bonds and can be stretched more than unfilled rubber [11]. The highest elongation at break was obtained for the 40 phr mDE filler. This could be due to the strong interconnecting filler–polymer networks, with fewer filler–filler networks below the filler percolation level [11]. Hybrid filler systems may have improved filler dispersion, and hybrid filler networks cause more effectively bonded rubber [62,63]. Strong rubber–filler interaction inhibits the extension of rubber chains and has a similar or reduced elongation at break values as unfilled rubber.

Figure 5f shows the variation in fracture toughness with the filler amount. From this figure, it is clear that the toughness of the composites can be enhanced by up to 40 phr of filler content. After 40 phr, the hybrid filler showed reduced fracture toughness, whereas the mDE showed only an insignificant change in toughness. Although the 60 phr hybrid filler system has a lower toughness value compared to 20 or 40 phr, it is still higher than the 3 phr MWCNT and unfilled rubber compounds. Toughness generally depends on the overall bonding strength, considering both the physical and chemical properties [64]. In a later section, it is observed that the cross-link densities of the rubber composites are optimised at an optimum filler concentration. In addition, optimum rubber–filler networks can only be achieved with an optimum amount of both filler and rubber. At higher filler amounts, the rubber fraction was reduced to obtain fewer rubber–filler networks, thereby reducing the fracture toughness. Owing to the better filler distribution and rubber–filler interactions, the fracture toughness of NR/37-mDE/3-CNT was 484% higher than that of the unfilled natural rubber.

**Figure 6:**
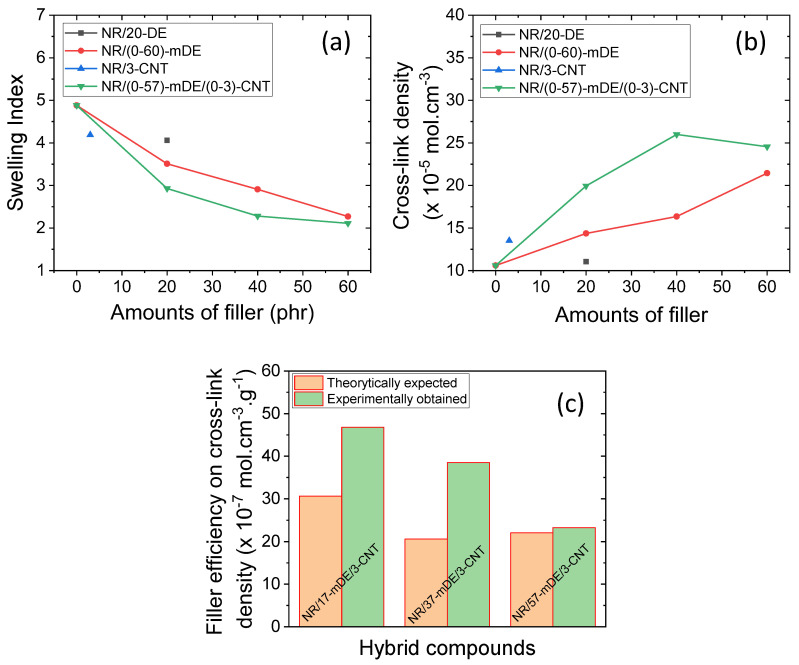
Swelling properties: (**a**) variation in swelling indexes with filler amounts, (**b**) variation in cross-link densities with filler amounts, and (**c**) filler efficiencies on cross-link densities to the hybrid filler systems.

The swelling and cross-linking characteristics of the rubber compounds are shown in Figure 6a–c. As shown in Figure 6a, the swelling index gradually decreases with increasing filler content. This suggests that an increase in the amount of filler enhanced the rubber–filler interactions. The hybrid filler showed a higher rate of decrease in the swelling index than the mDE-only filler system. Hybrid filler systems are expected to have higher surface areas than mDE filler systems at similar filler amounts and more interactions with rubber. From Figure 6b, it is evident that the composites containing the hybrid fillers showed higher cross-linking densities than the mDE filler systems with similar filler amounts. The highest cross-linking density was obtained for the 40 phr hybrid filler containing the NR/37-mDE/3-CNT composite. When the filler amount was greater than 40 phr, the cross-link density was slightly reduced, which may have been due to a significant reduction in the rubber fraction. Although the 40 phr hybrid filler shows the highest cross-link density, it does not merely indicate the highest toughness value but also roughly indicates a better modulus value compared to 20 phr. It is believed that because of the decrease in the rubber fraction at higher filler contents, particularly over filler percolation, the extensibility of rubber chains may be reduced, as is evident from the reduced elongation at break values.

Figure 6c shows the filler efficiencies of the hybrid filler systems used to investigate the synergism in the cross-linking density. The cross-linking efficiencies of the hybrid filler systems were determined by dividing the cross-linking densities by the filler amount. It can be seen that the experimentally obtained cross-link densities in the hybrid filler systems are much better than the theoretically calculated cross-link densities from the contributions of the individual fillers. This synergism in the cross-link densities of the hybrid filler systems may be due to the improved mDE filler distribution in the composites, which may have a greater effect on the curing activity [13].

**Figure 7:**
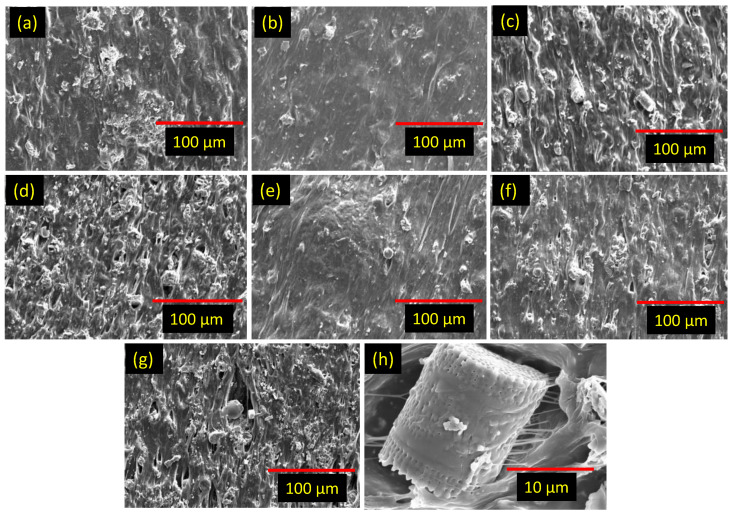
SEM images of rubber composites; (**a**) NR/20-DE, (**b**) NR/20-mDE, (**c**) NR/40-mDE, (**d**) NR/60-mDE, (**e**) NR/17-mDE/3-CNT, (**f**) NR/37-mDE/3-CNT, (**g**) NR/57-mDE/3-CNT, and (**h**) NR/57-mDE/3-CNT at higher resolution.

The improved mechanical and physical characteristics of the rubber composites can be further understood from a microscopic view of the filler distribution, as shown in Figure 7a–h. From Figure 7a,b, it is clear that the stearic acid modification of DE significantly improved the adhesion of rubber to the filler surface. From Figure 7b–d, it is clear that the rubber–filler compatibility can be reduced at higher filler amounts when mDE is used as a single filler system. Further improvement in the filler–rubber compatibility can be achieved with the addition of CNT in the hybrid filler systems (Figure 7e–g) compared to the mDE-filled composites (Figure 7b–d) at similar filler amounts. This may be due to the more homogeneous filler distribution in the hybrid filler systems during solvent-aided mixing [51]. Also, from Figure 7h, it can be seen that rubber molecules can enter the DE particles through the pores, and effective rubber–filler binding is possible. However, at higher filler amounts, the separation of the contact areas between the rubber and filler increased with strain (Figure 7h) and hence significantly reduced the elongation at break.

**Figure 8:**
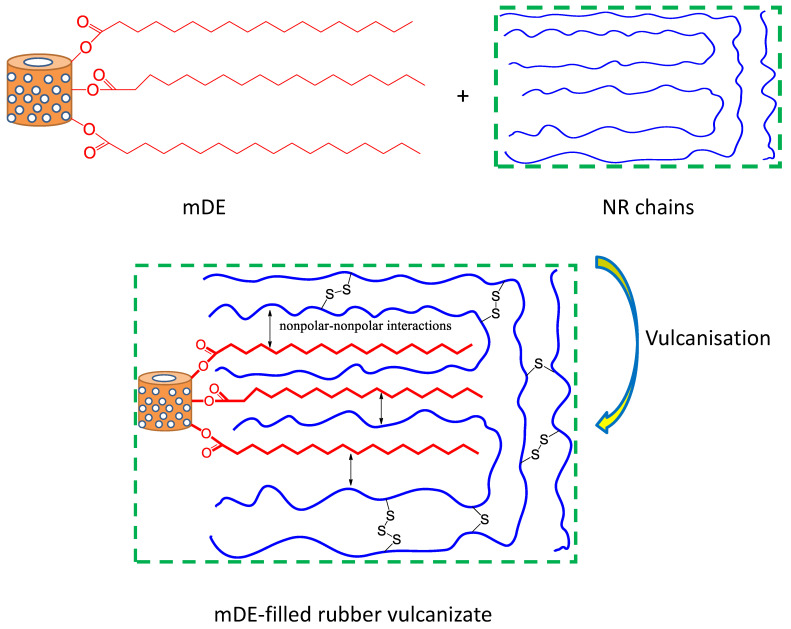
Possible reinforcing mechanism of rubber by stearic acid-modified diatomaceous earth.

From the study of filler modification and the observed mechanical properties, a simple mechanism can be drawn for the reinforcement of rubber using stearic-acid-modified DE, as shown in Figure 8. Stearic acid primarily forms strong bonds with DE through its polar groups. However, the non-polar tail may have interacted with the non-polar rubber chains through van der Waals-type interactions. Because stearic acid has long-chain hydrocarbons, the interactions become strong, and significant rubber reinforcement is possible.

### 3.3. Electrical and Energy Harvesting Performances of the Rubber Composites

**Figure 9:**
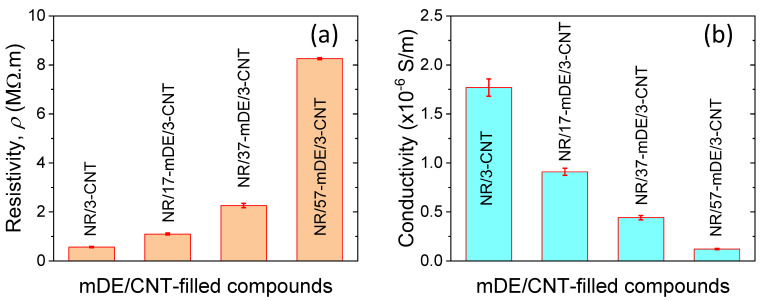
Electrical properties of mDE/CNT–filled rubber composites: (**a**) resistivity and (**b**) conductivity.

It is well known that only electrically conducting materials can improve the electrical properties of rubber composites. However, the choice of filler is an important factor in obtaining better electrical and mechanical properties. CNTs have a high surface area and aspect ratio, exhibit very good interactions with rubber, and can enhance their mechanical properties. The basic electrical properties, such as resistivity and conductivity, of the rubber composites, are shown in Figure 9a,b. From Figure 9a, it is evident that the resistivity increases almost linearly with an increase in the mDE content with a fixed amount of CNT in the rubber composites up to 40 phr of filler. Subsequently, it increased exponentially with an increase in the filler amount. This could be because of the percolation of more dielectric mDE fillers in the rubber composites [39]. Higher amounts of dielectric materials can enhance the dielectric properties of rubber and, hence, lower its conductivity, as shown in Figure 9b. Nevertheless, all rubber composites showed very good electrical conductivity in the semiconducting region.

**Figure 10:**
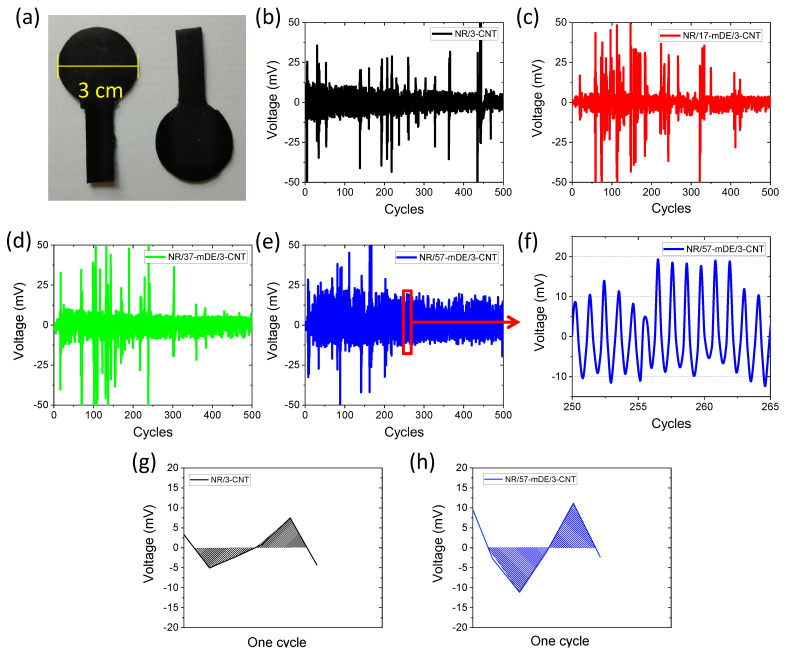
(**a**) Energy harvesting specimens, (**b**–**f**) voltage output with dynamic loading–unloading cycles for different rubber composites, and (**g**,**h**) areas in one cyclic loading–unloading.

The specimens used for the energy-harvesting study are shown in Figure 10a, and the energy-harvesting efficiencies are shown in Figure 10b–h. According to the capacitor network model [24,65], a large number of microcapacitors can be formed when a conducting material incorporates a dielectric material. It is also seen that these capacitors are sensitive to mechanical force and can act as transducers of mechanical force to electrical energy during the charging and discharging processes during the change in the capacitance value upon mechanical deformation [66,67]. The change in the capacitance of the composites with the loading–unloading cycles is evident from the output voltages. From Figure 10b–d, it is evident that the output voltages are sometimes much higher than the regular voltages. It is believed that upon the addition of conducting materials (CNT), microcapacitors have different capacitance values depending on the distance between the conducting and non-conducting paths, assuming a parallel-plate-type capacitor. Upon the application of force, a small change in the distance can change the large voltage gradient for capacitors with higher capacitance values. The capacitor exhibits a higher capacitance at the minimum distance between the conducting and non-conducting paths. Below this critical distance, the two electrodes may collapse and lose their capacitance and sensitivity drift in the next cycle. However, these incidents are irregular because the conducting paths are unstable in viscoelastic materials [68,69]. Some new conducting paths were generated, and some were destroyed by the applied deformation. As shown in Figure 10b–e, increasing the mDE content enhanced the intensities of the regular peaks, and the NR/57-mDE/3-CNT composites showed very regular peaks with higher intensities after 250 loading–unloading cycles. This could be because of the generation of the highest number of microcapacitors at the electrical percolation threshold and uniform capacitance values through uniform dispersion of CNT in the rubber matrix. Figure 10f shows regular peaks between −10 and +10 mV, which is sufficient for sensing very low mechanical deformations because modern electronic devices can measure as low as microvolts. It is evident that the energy output of the NR/57-mDE/3-CNT composite is nearly double that of the CNT-filled composite, considering the representative areas per cycle in Figure 10g,h. Because of their robust mechanical and energy-conversion efficiencies, mDE and CNT hybrid filler-based composites may find suitable applications in electromechanical energy harvesting and other mechanical sensing devices.

## 4. Conclusions

This study presents the successful development of rubber composites based on naturally available diatomaceous earth with successful filler modification by stearic acid using a convenient method. The FT-IR study and the mechanical properties of the rubber composites suggest that stearic acid effectively modifies the diatomaceous earth. Compared to pristine diatomaceous earth, stearic acid-modified diatomaceous earth exhibited better reinforcing properties. Modified diatomaceous earth can be useful up to 60 phr, with improved mechanical properties compared with unfilled rubber. Hybrid fillers comprising modified diatomaceous earth and multi-walled carbon nanotubes in suitable amounts can further improve the mechanical and electrical properties of rubber composites. For example, a composite based on a hybrid filler of 37 phr mDE and 3 phr CNT has higher tensile strength and fracture toughness values of around 429% and 484%, respectively, in comparison to the unfilled rubber vulcanisate. Hybrid fillers comprising modified diatomaceous earth and CNT improved the energy-harvesting efficiencies of rubber composites at higher mDE filler amounts. Thus, the fabricated rubber composites can be useful for harvesting electrical power from renewable mechanical energy sources and for electromechanical sensing applications. Currently, the hybrid composite can be commercialised for low mechanical energy purposes due to its low fracture toughness value at higher mDE content. However, the fracture toughness and mechanical stability can be further improved by employing a certain amount of reinforcing piezoelectric nanomaterial, a step that will be undertaken in future extended work.

## Figures and Tables

**Table 1:** Mixing composition for rubber composites per hundred grams of rubber (phr).

Formulations	Masterbatch	Amounts of DE/mDE (phr)	Amounts of CNT (phr)
NR-unfilled	100	-	-
NR/20-DE	100	20	-
NR/20-mDE	100	20	-
NR/40-mDE	100	40	-
NR/60-mDE	100	60	-
NR/3-CNT	100	-	3
NR/17-mDE/3-CNT	100	17	3
NR/37-mDE/3-CNT	100	37	3
NR/57-mDE/3-CNT	100	57	3

## Data Availability

Data will be available upon request to the corresponding author.
